# Effects and Moderators of Exergaming on Glucose and Lipid Metabolism in Children and Adolescents: Systematic Review and Meta-Analysis

**DOI:** 10.2196/93610

**Published:** 2026-09-15

**Authors:** Shuo Zhou, Yang Yang, Xiaoquan Weng, Huakun Zheng, Lingyan Yuan

**Affiliations:** 1 School of Physical Education, Shanghai Normal University, No. 100, Haisi Road, Fengxian District, Shanghai, 201418, China, 86 13918061251; 2 School of Exercise and Health, Shanghai University of Sport, Shanghai, China

**Keywords:** Exergaming, active video games, glucose metabolism, lipid metabolism, children and adolescents, meta-analysis

## Abstract

**Background:**

Exergaming, or active video gaming, may make physical activity more engaging for children and adolescents. Earlier reviews have focused on activity, adiposity, fitness, or psychosocial outcomes, leaving effects on glycemic and lipid biomarkers uncertain. Their cardiometabolic relevance warrants synthesis.

**Objective:**

This systematic review and meta-analysis evaluated the effects of exergaming on glucose and lipid metabolism in children and adolescents, and explored potential moderators.

**Methods:**

Following PRISMA 2020 (Preferred Reporting Items for Systematic Reviews and Meta-Analyses), we searched PubMed, Embase, Scopus, Web of Science, the Psychology and Behavioral Sciences Collection, and CENTRAL from inception to April 27, 2026, with backward and forward citation searching. Eligible controlled interventions enrolled participants aged ≤18 years and compared exergaming with nonexergaming controls. Outcomes were glucose, insulin, total cholesterol (TC), triglycerides, high-density lipoprotein cholesterol (HDL-C), and low-density lipoprotein cholesterol (LDL-C). Standardized mean differences (Hedges *g*) were pooled with inverse-variance random-effects models and the Hartung-Knapp-Sidik-Jonkman adjustment. We reported 95% CIs, heterogeneity, 95% prediction intervals (PIs), risk of bias, and GRADE (Grading of Recommendations Assessment, Development and Evaluation) certainty; subgroup analysis, meta-regression, and leave-one-out sensitivity analyses explored moderators and estimate stability.

**Results:**

Ten controlled intervention studies involving 732 participants were included. Pooled effects were nonsignificant for glucose (9 studies, 528 participants; Hedges *g*=−0.16, 95% CI −0.57 to 0.24; PI −1.12 to 0.79), insulin (5 studies, 308 participants; Hedges *g*=−0.06, 95% CI −0.35 to 0.23; PI −0.39 to 0.27), TC (9 studies, 510 participants; Hedges *g*=−0.39, 95% CI −1.11 to 0.33; PI −1.70 to 0.93), triglycerides (9 studies, 510 participants; Hedges *g*=−0.17, 95% CI −0.50 to 0.17; PI −0.88 to 0.55), and HDL-C (9 studies, 510 participants; Hedges *g*=−0.14, 95% CI −0.34 to 0.06; PI −0.35 to 0.07; effect sizes were reverse-oriented so that negative values favored exergaming). LDL-C showed a small favorable pooled average reduction (9 studies, 510 participants; Hedges *g*=−0.29, 95% CI −0.55 to −0.04; PI −0.73 to 0.14), but its PI crossed the null. All PIs crossed the null; heterogeneity was substantial for glucose, considerable for TC, moderate for triglycerides, and low for insulin, HDL-C, and LDL-C. Subgroup signals were exploratory, no significant meta-regression moderators were identified, and leave-one-out analyses did not materially alter the glucose, TC, or triglycerides estimates. Most randomized trials had risk-of-bias concerns; the nonrandomized study had serious risk of bias. These limitations reduce confidence in the LDL-C signal. GRADE certainty was very low for glucose and TC and low for the remaining outcomes.

**Conclusions:**

This review provides a synthesis of pediatric glycemic and lipid biomarkers and potential moderators, extending earlier broader exergaming reviews. Exergaming may be an engaging complementary strategy for physical activity and lipid health, including LDL-C, in schools, families, and community settings. However, heterogeneous protocols, risk-of-bias concerns, very low-to-low certainty, and PIs crossing the null mean that the magnitude and generalizability of any metabolic benefit remain uncertain.

## Introduction

Child and adolescent obesity remains a major public health concern worldwide [1,2], and it is closely related to abnormalities in glucose and lipid metabolism during growth and development [3,4]. Insufficient physical activity is widespread among adolescents [5], and excessive screen-based sedentary behavior may further contribute to cardiometabolic risk [6,7]. Early metabolic dysregulation may adversely affect childhood health and increase the risk of cardiovascular disease and type 2 diabetes later in life [8-10]. Therefore, interventions for pediatric obesity and metabolic health should not focus only on weight control, but should also target modifiable metabolic risk markers, including glucose, insulin, and lipid profiles [4]. Although conventional school- and community-based exercise programs can be beneficial, their implementation is often limited by low motivation, poor adherence, and limited environmental support [8,11]. More engaging and scalable physical activity strategies are therefore needed for children and adolescents.

Exergaming, also referred to as active video gaming, combines digital gameplay with bodily movement and has emerged as a promising strategy for promoting physical activity in young people [12-14]. Through motion-based interaction, immediate feedback, goal setting, and reward mechanisms, exergaming may transform part of sedentary screen time into active movement and increase enjoyment and adherence to exercise [13-17]. Previous studies and reviews suggest that exergaming can increase energy expenditure and may improve selected health-related outcomes, such as body composition and physical fitness [18-23]. Recent advances in motion-capture systems, AI, and virtual reality (VR) have further expanded the potential application of exergaming in pediatric health promotion [15]. However, whether exergaming produces consistent improvements in glucose- and lipid-related metabolic biomarkers remains uncertain.

The existing evidence is limited in several important respects. First, individual trials have reported mixed findings, with some showing improvements in metabolic indicators and others reporting small or nonsignificant effects [23-25]. These inconsistencies may reflect differences in exergaming modality, exercise intensity, intervention duration, session frequency, comparator type, and participant characteristics, including age, sex, weight status, and baseline metabolic risk [14,24,26]. Second, previous syntheses of exergaming or active video games in youth have primarily emphasized physical activity, body composition, obesity, fitness, enjoyment, or broad cardiometabolic outcomes rather than prespecified glucose and lipid biomarkers [19,20,23]. Third, the potential moderators of metabolic responses to exergaming remain unclear. In particular, it is not yet well established which type of exergaming, intervention dose, or participant subgroup is most likely to benefit. Even in broader clinical populations, game-based and interactive exercise interventions have shown heterogeneous cardiometabolic effects, highlighting the need for more focused and moderator-informed evidence in pediatric populations [25,27].

To address these gaps, this systematic review and meta-analysis evaluated the effects of exergaming on glucose and lipid metabolism in children and adolescents compared with control conditions. Specifically, we pooled effect sizes for glucose, insulin, total cholesterol (TC), triglycerides, high-density lipoprotein cholesterol, and low-density lipoprotein cholesterol. We also examined heterogeneity, reported prediction intervals (PIs) to distinguish pooled average effects from the range of effects expected in future settings, rated certainty using GRADE (Grading of Recommendations Assessment, Development and Evaluation), and conducted subgroup analyses and exploratory meta-regression for intervention and participant characteristics. This focus distinguishes the present review from prior exergaming reviews by treating glycemic and lipid biomarkers as primary outcomes and by examining whether intervention or participant characteristics may moderate metabolic responses. This review is timely because recent VR-based, home-based, adaptive, and narrative-enhanced exergaming trials have expanded the evidence base and may differ from earlier commercial console interventions in intensity, supervision, and delivery context.

## Methods

### Reporting Standard and Registration

This systematic review was reported in accordance with the PRISMA (Preferred Reporting Items for Systematic Reviews and Meta-Analyses) 2020 statement [28]. The completed PRISMA 2020 checklist is provided in Checklist 1. The review protocol was registered in the Open Science Framework.

### Information Sources

We systematically searched PubMed, Embase, Scopus, Web of Science, the Psychology and Behavioral Sciences Collection (the Psychology and Behavioral Sciences Collection via EBSCOhost), and the CENTRAL from database inception to April 27, 2026. Backward citation searching was conducted by screening the reference lists of all included studies and relevant reviews. Forward citation searching was additionally performed using citation tracking in Web of Science, Scopus, and Google Scholar to identify studies that cited the included trials.

### Search Strategy

A search strategy was developed to identify studies on exergaming interventions in children and adolescents and their effects on glucose- and lipid-related metabolic outcomes. The search process was reported with reference to the PRISMA-S extension [29]. The main searches combined exergaming-related terms and pediatric population terms, using both controlled vocabulary (eg, MeSH and Emtree where applicable) and free-text keywords, with syntax adapted for each database. To reduce the risk of missing studies in which metabolic outcomes were reported as secondary outcomes, the main database searches did not require glucose- or lipid-related terms to be present in the title, abstract, or indexing fields of all records. Full search strategies for all databases, including database-specific syntax, field tags, controlled vocabulary terms, and free-text terms, are provided in Multimedia Appendix 1.

### Selection Process

All retrieved records were imported into EndNote X9 (Clarivate) for reference management, and duplicate records were removed before screening. Two reviewers (ZS and ZHK) independently screened titles and abstracts against the predefined eligibility criteria. Full texts of potentially eligible studies were then assessed independently by the same 2 reviewers, and reasons for exclusion at the full-text stage were recorded. Disagreements at either stage were resolved through discussion and reexamination of the eligibility criteria; when consensus could not be reached, a third reviewer (WXQ) adjudicated the decision.

### Eligibility Criteria

Study selection was guided by the PICOS (Population, Intervention, Comparison, Outcomes, and Study design) framework. Studies were eligible if they met all of the following criteria: (1) population: children or adolescents aged 18 years or younger, consistent with the Convention on the Rights of the Child definition of childhood as under 18 years of age [30], from generally healthy or overweight or obesity pediatric populations, without specific chronic or special disease conditions; (2) intervention: exergaming or active video game-based exercise interventions (eg, Wii or Wii Fit; Nintendo Co Ltd, Kinect; Microsoft Corp, Dance Dance Revolution; Konami Digital Entertainment Co Ltd, GameBike; CatEye Co Ltd, or virtual reality–based exergaming), delivered either as an acute single-bout session or as a chronic multiweek intervention, in which physically active gameplay was the primary exercise modality; (3) comparison: an eligible comparator condition, including usual care, no intervention, wait-list, seated rest, sedentary gaming, or other nonexergaming conditions; (4) outcomes: at least 1 glucose- or lipid-related metabolic outcome, including glucose, insulin, TC, triglycerides, high-density lipoprotein cholesterol (HDL-C), or low-density lipoprotein cholesterol (LDL-C), with sufficient numerical data available for quantitative synthesis; and (5) study design and publication type: peer-reviewed full-text controlled intervention studies, including randomized and nonrandomized designs and parallel-group or crossover studies.

Studies were excluded if they were nonoriginal or nonpeer-reviewed reports (eg, conference abstracts, protocols, trial registrations, reviews, editorials, or letters), included adult-only samples or pediatric populations with specific chronic or special diseases, did not involve an eligible exergaming intervention, did not report prespecified glucose- or lipid-related outcomes, or lacked sufficient numerical data for quantitative synthesis.

### Data Extraction

Data extraction was performed independently by 2 reviewers (ZS and ZHK) using a piloted Excel-based data extraction form that was finalized before full-text review. Extracted information included study identification, country, study design, participant characteristics, intervention and comparator characteristics, intervention duration and frequency, exergaming mode, and outcome data. A third reviewer (WXQ) conducted an additional round of checking. Disagreements were resolved through discussion, and a fourth reviewer (YY) was consulted when consensus could not be reached. If outcome data were missing or presented only graphically, the study authors were contacted to request the necessary information. If numerical data could not be obtained from the authors and remained available only in graphical form, relevant data were extracted using WebPlotDigitizer version 4.7 (Ankit Rohatgi) [31]. Studies lacking sufficient numerical data were excluded from quantitative synthesis. For each eligible outcome, the mean (SD) and sample size were extracted for each group at baseline and postintervention, or as change scores when reported.

### Data Conversion

We extracted the mean (SD) and sample size reported for each condition and outcome. If the study only reported confidence intervals, they were converted to SD using the following formula [32]:


$$\text{SD}\text{=}\sqrt{\text{N}}\frac{{\text{CI}}_{\text{h}\text{ig}\text{h}}\text{− }{\text{CI}}_{\text{low}}}{\text{2}\text{t}}$$

where CI_high_ is the upper limit of the CI, CI_low_ is the lower limit of the CI, and t is the t distribution value with N−1 degrees of freedom for the respective confidence level [32].

If the study only reported SEs, they were converted to SD using the following formula [32]:


$$\text{SD}\text{=}\sqrt{\text{N}}\text{ ×}\text{SE}$$

### Risk-of-Bias Assessment

Risk of bias was assessed independently by 2 reviewers (ZS and ZHK). For randomized controlled trials, the Cochrane Risk of Bias 2 (RoB 2) tool was used [33], and for the nonrandomized study, the Risk Of Bias In Nonrandomized Studies of Interventions (ROBINS-I) tool was applied [34]. Any disagreements were resolved through discussion, and when necessary, consultation with a third reviewer (WXQ).

The RoB 2 tool evaluates bias in 5 domains: bias arising from the randomization process, bias due to deviations from intended interventions, bias due to missing outcome data, bias in measurement of the outcome, and bias in selection of the reported result [33]. The ROBINS-I tool evaluates bias due to confounding, selection of participants, classification of interventions, deviations from intended interventions, missing data, measurement of outcomes, and selection of the reported result [34].

In addition, the methodological quality of randomized controlled trials was assessed descriptively using the Physiotherapy Evidence Database (PEDro) scale [35]. The PEDro scale contains 11 items, of which the first item relates to external validity and is not included in the total score; therefore, the total score ranges from 0 to 10, with higher scores indicating better methodological quality [35]. PEDro scores were used as a supplementary descriptive assessment and were not used to exclude studies, weight effect estimates, or determine the certainty of evidence.

### Effect Measures and Data Synthesis

Quantitative synthesis was performed using random-effects meta-analysis with the inverse-variance method [32,36,37]. Because outcome measures were reported using different units across studies, standardized mean differences were calculated as Hedges *g* with small-sample correction [38]. Hedges *g* values of 0.2, 0.5, and 0.8 were interpreted as small, moderate, and large effects, respectively [39]. For each eligible outcome, effect sizes were calculated from the available numerical data using a prespecified and consistent approach across studies. Postintervention group means and SDs were preferentially used to calculate effect sizes; when only change-score data were reported, these were used instead. To make clinical interpretation consistent across outcomes and forest plots, effect sizes were oriented so that negative values favored exergaming. For high-density lipoprotein cholesterol (HDL-C), only the direction of the effect size was reversed for meta-analytic interpretation because higher HDL-C is clinically favorable; original group means and SDs were not changed.

For multiarm trials with more than one eligible exergaming intervention arm sharing a single control group, eligible exergaming arms were combined into a single intervention group before pairwise meta-analysis to avoid double counting of the control group. Combined means and SDs were calculated according to the formulas recommended in the Cochrane Handbook [32]. Intervention arms that did not meet the eligibility criteria for the exergaming-vs-control comparison were not included in the pooled analysis.

Random-effects models were fitted using the inverse-variance method, and the Hartung-Knapp-Sidik-Jonkman adjustment (Knapp-Hartung adjustment) was applied to the CIs and statistical tests of pooled effects [32,40]. Pooled effects were presented with 95% CIs. Where sufficient studies were available, 95% PIs were calculated to estimate the range of true effects expected in comparable future settings and were interpreted alongside heterogeneity and GRADE certainty [41].

Statistical heterogeneity was assessed using Cochran Q, *I*², and *τ*² [32]. *I*² values of 0%‐25%, 25%‐50%, 50%‐75%, and 75%‐100% were interpreted as indicating low, moderate, substantial, and considerable heterogeneity, respectively, together with clinical and methodological considerations [32]. Statistical analyses and visualizations were performed in R version 4.2.0 (R Foundation for Statistical Computing) using the meta and metafor packages [42]. Statistical significance was set at *P*<.05.

### Subgroup and Meta-Regression Analyses

To explore potential sources of between-study heterogeneity, prespecified subgroup analyses were conducted for categorical moderators where sufficient studies were available [43]. Because most outcome-specific meta-analyses in this review included fewer than 10 studies, subgroup findings were considered exploratory and interpreted cautiously [32,44]. The following variables were examined in subgroup analyses where feasible: (1) age group, (2) sex composition, (3) weight status, (4) exergaming mode, and (5) intervention type. Age was categorized using a 12-year cut point (children: ≤12 years; adolescents: ≥13 years) for operational comparability across studies [45,46]. Sex composition was classified as mixed-sex, male-only, and female-only samples. Weight status was categorized as normal weight vs overweight or obesity, as defined by each included study. Exergaming mode was classified as commercial exergaming, customized research-developed exergaming, and VR-based exergaming. Intervention type was categorized as acute single-bout interventions vs chronic multiweek intervention programs.

Where sufficient studies were available, univariable random-effects meta-regression analyses were performed in R using the metafor package [42] to examine continuous moderators, including baseline BMI, single-session duration (minutes), total intervention duration (weeks), intervention frequency (sessions/week), and mean participant age (years). Given the limited number of studies contributing to most outcomes, all meta-regression findings were considered exploratory [47].

### Reporting Bias and Sensitivity Analyses

Because all outcome-specific meta-analyses included fewer than 10 studies, we did not perform the Egger test or other formal statistical tests for funnel plot asymmetry, as these methods are underpowered and may be misleading when the number of studies is small [32,48-51]. Sensitivity analyses were prespecified to evaluate the robustness of pooled effect estimates. A leave-one-out approach was implemented, whereby each study was sequentially removed and the meta-analysis was repeated to assess whether any single study disproportionately influenced the overall findings. Outlier and influence diagnostics were also examined where appropriate [52]. For each iteration, the standardized mean difference (Hedges *g*), its 95% CI, and heterogeneity indices (*I*² and τ²) were recalculated to evaluate the stability of the pooled estimates.

### Certainty of the Evidence

The certainty of the evidence for each outcome was assessed using the GRADE approach [53]. Certainty was rated as high, moderate, low, or very low after explicit consideration of risk of bias, inconsistency, indirectness, imprecision, and publication bias [53]. An official GRADEpro GDT (Guideline Development Tool) Summary of Findings table was prepared for all six outcomes and reports the number and design of contributing studies, standardized mean differences with 95% CIs, certainty ratings, and concise reasons for downgrading.

All GRADE assessments were completed by one reviewer (ZS) and checked by a second reviewer (ZHK), with disagreements resolved through discussion.

## Results

### Study Selection

**Figure 1.**
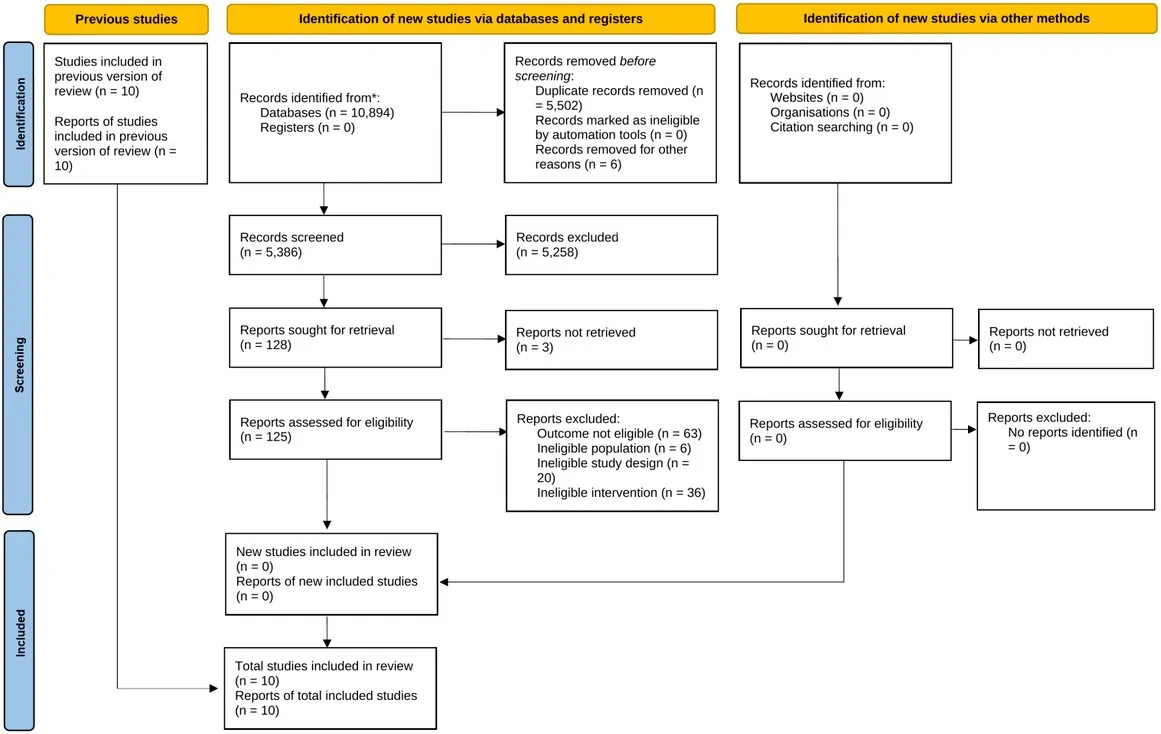
PRISMA (Preferred Reporting Items for Systematic Reviews and Meta-Analyses) 2020 flow diagram for a new systematic review that included searches of databases, registers, and other sources.

The database search conducted on April 27, 2026, identified 10,894 records across 6 databases. After removal of 5502 duplicate records and 6 records for other reasons, 5386 records were screened by title and abstract. Of these, 5258 records were excluded, and 128 reports were sought for retrieval. Three reports could not be retrieved, leaving 125 full-text reports assessed for eligibility. All 125 reports identified through database searching were excluded: 63 did not report an eligible outcome, 6 had an ineligible population, 20 had an ineligible study design, and 36 used an ineligible intervention. Backward and forward citation searching did not identify additional eligible studies. The final review therefore included 10 studies for quantitative synthesis (Figure 1).

### Characteristics of Included Studies

Table 1 summarizes the characteristics of the included studies. A total of 10 studies involving 732 participants were included [54-63]. The studies were conducted in the United States (n=4), South Korea (n=2), and China, Tunisia, Canada, and the United Kingdom (n=1 each). Most studies enrolled children or adolescents with overweight or obesity, although 1 study included adolescents with excess body weight and 1 acute study included boys without excess body weight. The interventions included VR-based sports systems, active video games, school-based Wii programs, dance exergaming, home-based console exergaming, and interactive video game cycling. Eight studies [55,56,58-63] evaluated chronic interventions lasting 4 to 24 weeks, whereas 2 studies [54,57] used acute single-session designs. The most commonly assessed metabolic outcomes were glucose, insulin, TC, triglycerides, HDL-C, and LDL-C.

**Table 1.** Characteristics of included studies [54-63].

Author, year	Country	Sample size (N)	Age (in years)	Sex distribution n (male; female)	BMI (kg/m²)	Intervention	Duration	Measurement
Wang et al, 2025 [58]	China	N=240 IG1^a^ IG2 CG^b^ (96/96/48)	14.2	(162; 78)	27.13	IG1: adaptive AI-based VR^c^ sports system (REVERIE^d^; VR table tennis and VR soccer) IG2: real-world sports training (table tennis and soccer) CG: regular physical education classes	45 min/session 3 sessions/week 8 weeks	Glucose, insulin, TC^e^, triglycerides, HDL-C^f^, LDL-C^g^
Lu et al, 2025 [59]	United States	N=135 IG1 IG2 CG (47/48/40)	10.9	NR^h^	27.21	IG1: narrative-enhanced active video game IG2: active video game CG: advised to complete at least 60 min of physical activity weekly	30‐60 min/session 7 sessions/week 24 weeks	Glucose, insulin, TC, triglycerides, HDL-C, LDL-C
Abedelmalek et al, 2022 [60]	Tunisia	N=32 (16/16)	16	(32; 0)	35.77	IG: cooperative sport exergaming CG: no additional exercise	45 min/session 5 sessions/week 4 weeks	TC, triglycerides, HDL-C, LDL-C
Chae et al, 2022 [61]	South Korea	N=127 (60/67)	15.84	NR	22.06	IG: school-based “We Fit” weight control program using Nintendo Wii Sports CG: usual school routine	30 min/session 5 sessions/week 12 weeks	Glucose, TC, triglycerides, HDL-C, LDL-C
Staiano et al, 2018 [62]	United States	N=46 (23/23)	11.2	(23; 23)	25.56	IG: home-based exergaming intervention (GameSquad; console-based exergames plus telehealth coaching) CG: usual care	60 min/session 3 sessions/week 24 weeks	Glucose, TC, triglycerides, HDL-C, LDL-C
Staiano et al, 2017 [63]	United States	N=41 (22/19)	15.5	(0; 41)	37.5	IG: dance exergaming (Just Dance; Dance Central) CG: usual care	60 min/session 3 sessions/week 12 weeks	Glucose, insulin, TC, triglycerides, HDL-C, LDL-C
Allsop et al, 2016 [57]	United Kingdom	N=22	9.9	(22; 0)	18.1	IG: active video gaming CG: seated video gaming	Single acute session 90 min	Glucose
Park et al, 2015 [54]	South Korea	N=24 (12/12)	14.83	(16; 8)	25	IG: acute active video gaming CG: seated rest after a high-fat meal	Single acute session 60 min	Glucose, TC, triglycerides, HDL-C, LDL-C
Adamo et al, 2010 [55]	Canada	N=30 (15/15)	14.5	NR	37.4	IG: interactive video game cycling (GameBike) CG: stationary cycling to music	60 min/session 2 sessions/week 10 weeks	Glucose, insulin, TC, triglycerides, HDL-C, LDL-C
Murphy et al, 2009 [56]	United States	N=35 (23/12)	10.21	(18; 17)	29.85	IG: Dance Dance Revolution–based exergaming CG: usual care	10‐30 min/session 5 sessions/week 12 weeks	Glucose, insulin, TC, triglycerides, HDL-C, LDL-C

a IG: intervention group.

b CG: control group.

c VR: virtual reality.

d REVERIE: real-world exercise and VR-based exercise research in education.

e TC: total cholesterol.

f HDL-C: high-density lipoprotein cholesterol.

g LDL-C: low-density lipoprotein cholesterol.

h NR: not reported.

### Primary Outcomes

**Figure 2.**
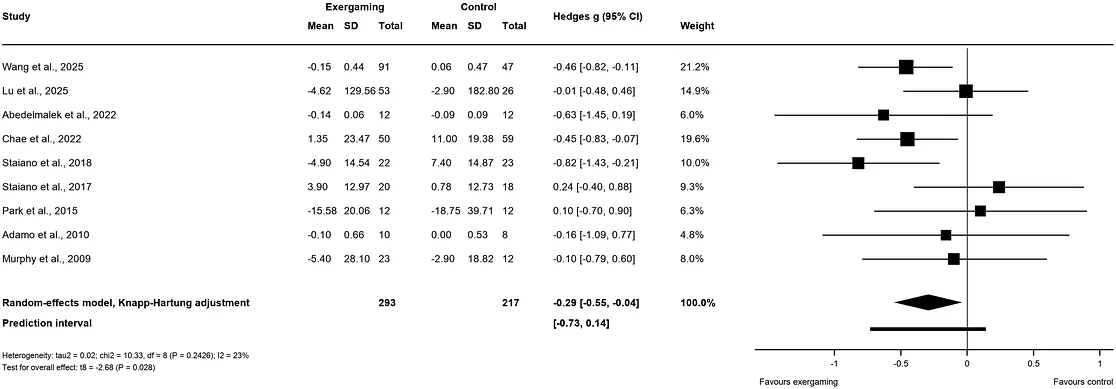
Forest plot of exergaming vs control for low-density lipoprotein cholesterol [54-56,58-63].

In the Hartung-Knapp-Sidik-Jonkman-adjusted random-effects meta-analyses with correction for double counting in multiarm trials, exergaming was associated with a small average reduction in LDL-C (9 studies, 510 participants; Hedges *g*=−0.29, 95% CI −0.55 to −0.04; *P*=.028; *I*²=23%; 95% PI −0.73 to 0.14; Figure 2). Although the pooled average effect favored exergaming, the PI crossed the null.

No significant pooled effects were observed for glucose (9 studies, 528 participants; Hedges *g*=−0.16; 95% CI −0.57 to 0.24; *P*=.381; *I*²=65%; 95% PI −1.12 to 0.79), insulin (5 studies, 308 participants; Hedges *g*=−0.06; 95% CI −0.35 to 0.23; *P*=.584; *I*²=0%; 95% PI −0.39 to 0.27), TC (9 studies, 510 participants; Hedges *g*=−0.39; 95% CI −1.11 to 0.33; *P*=.250; *I*²=77%; 95% PI −1.70 to 0.93), triglycerides (9 studies, 510 participants; Hedges *g*=−0.17; 95% CI −0.50 to 0.17; *P*=.292; *I*²=49%; 95% PI −0.88 to 0.55), or HDL-C, with effect sizes reverse-oriented so that negative values favored exergaming (9 studies, 510 participants; Hedges *g*=−0.14; 95% CI −0.34 to 0.06; *P*=.148; *I*²=0%; 95% PI −0.35 to 0.07).

**Figure 3.**
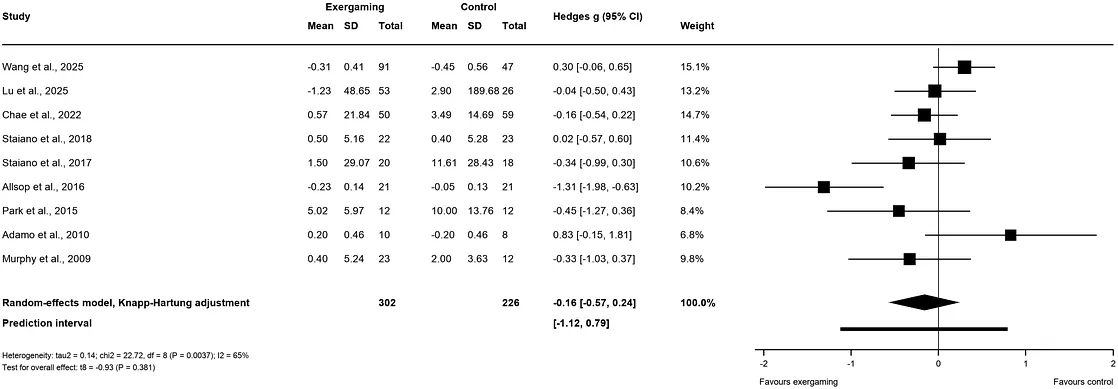
Forest plot of exergaming vs control for glucose [54-59,61-63].

**Figure 4.**
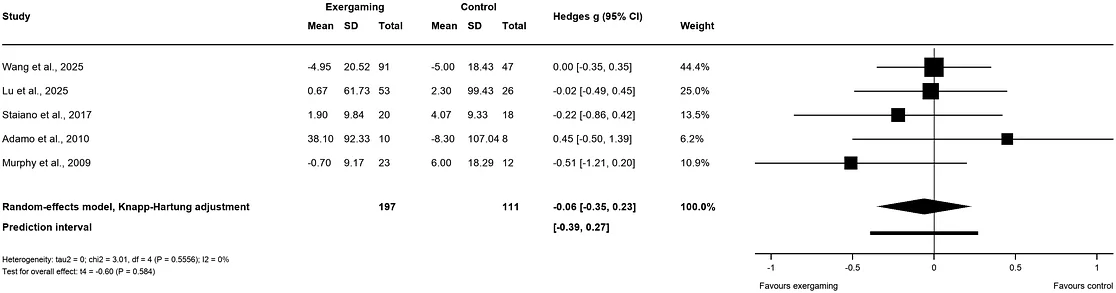
Forest plot of exergaming vs control for insulin [55,56,58,59,63].

**Figure 5.**
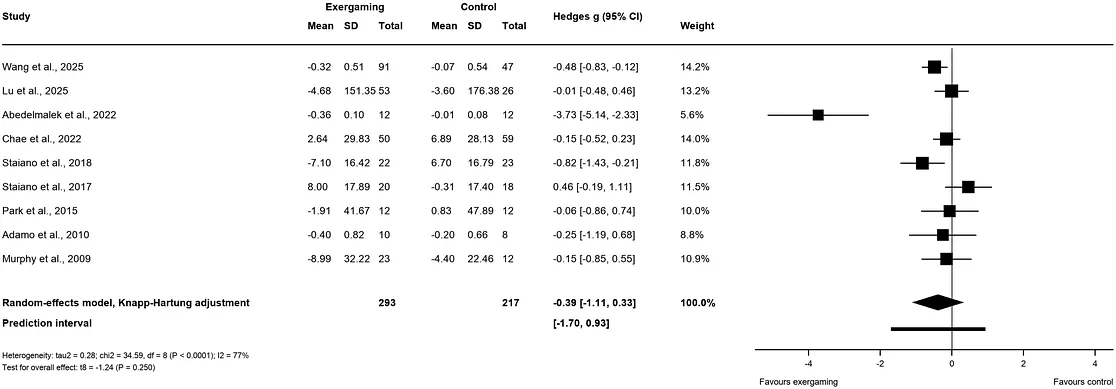
Forest plot of exergaming vs control for TC [54-56,58-63].

**Figure 6.**
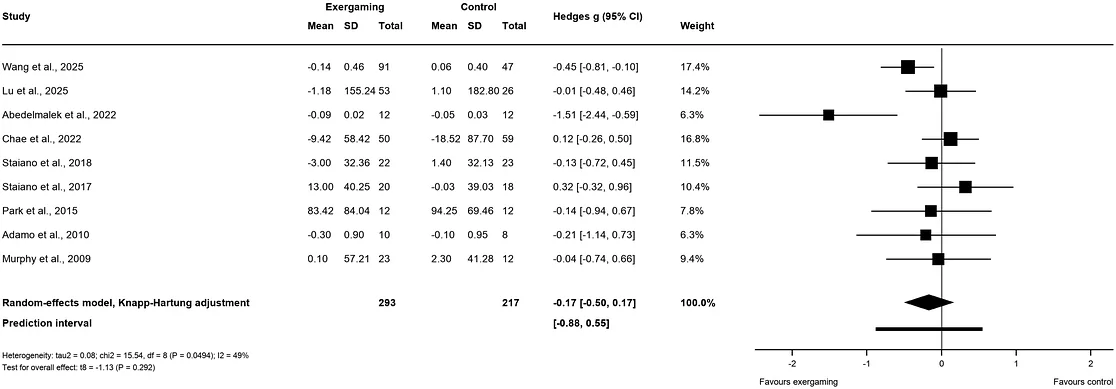
Forest plot of exergaming vs control for triglycerides [54-56,58-63].

**Figure 7.**
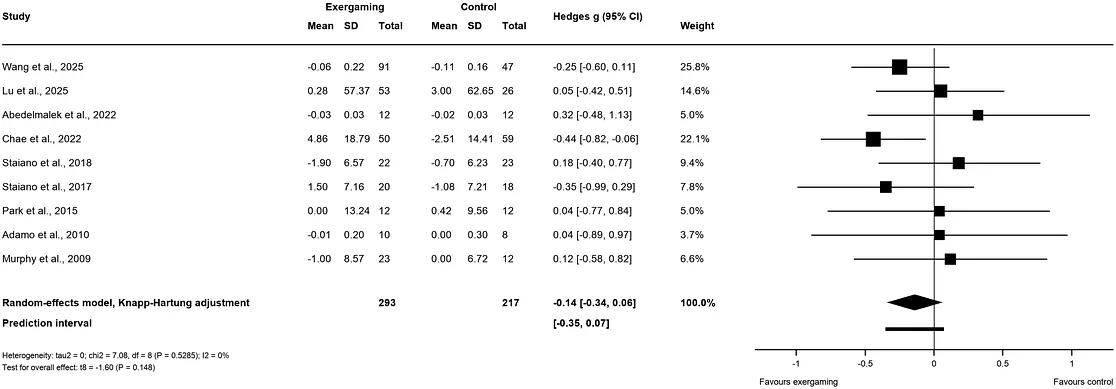
Forest plot of exergaming vs control for high-density lipoprotein cholesterol [54-56,58-63].

Heterogeneity was substantial for glucose and considerable for TC, moderate for triglycerides, and low for insulin, HDL-C, and LDL-C. All 95% PIs crossed the null, indicating that the average effects may vary across future settings. The glucose control-group SD of 189.68 for Lu et al (2025) [59] was verified against the original article and extraction file and was retained because it reflected the variability derived from the trial’s reported standard-error information. The outcome-specific forest plots are shown in Figures 2-7.

### Subgroup and Meta-Regression Analyses

Subgroup analyses were conducted according to sex, intervention type, exergaming modality, weight status, and age group (Multimedia Appendix 2). For glucose, significant between-subgroup differences were observed by sex (P_between=.001) and intervention type (P_between=.04), whereas no significant subgroup differences were found by exergaming modality (P_between=.07), weight status (P_between=.23), or age group (P_between=.27). The male-only subgroup showed a significant reduction in glucose (Hedges *g*=−1.31; 95% CI −1.98 to −0.63); however, this estimate was derived from a single study and should therefore be interpreted with caution. Acute interventions showed a larger negative effect estimate than chronic interventions, but the within-subgroup effect was not statistically significant and the confidence interval was wide, indicating substantial imprecision.

Between-subgroup differences were observed for TC by sex (P_between<.001), triglycerides by sex (P_between=.005) and exergaming mode (P_between=.03), and HDL-C by age group (P_between=.001). No significant subgroup differences were detected for LDL-C or insulin (all P_between>.05). LDL-C showed reductions across most subgroup analyses, but no between-subgroup test was significant. All subgroup findings were exploratory and should be interpreted cautiously because some categories contained only 1 or 2 studies.

Exploratory meta-regression analyses were performed for mean age, baseline BMI, weekly session frequency, total intervention duration, and session duration. No significant associations were observed between these study-level characteristics and pooled effect sizes (Multimedia Appendix 3).

### Risk of Bias

**Figure 8.**
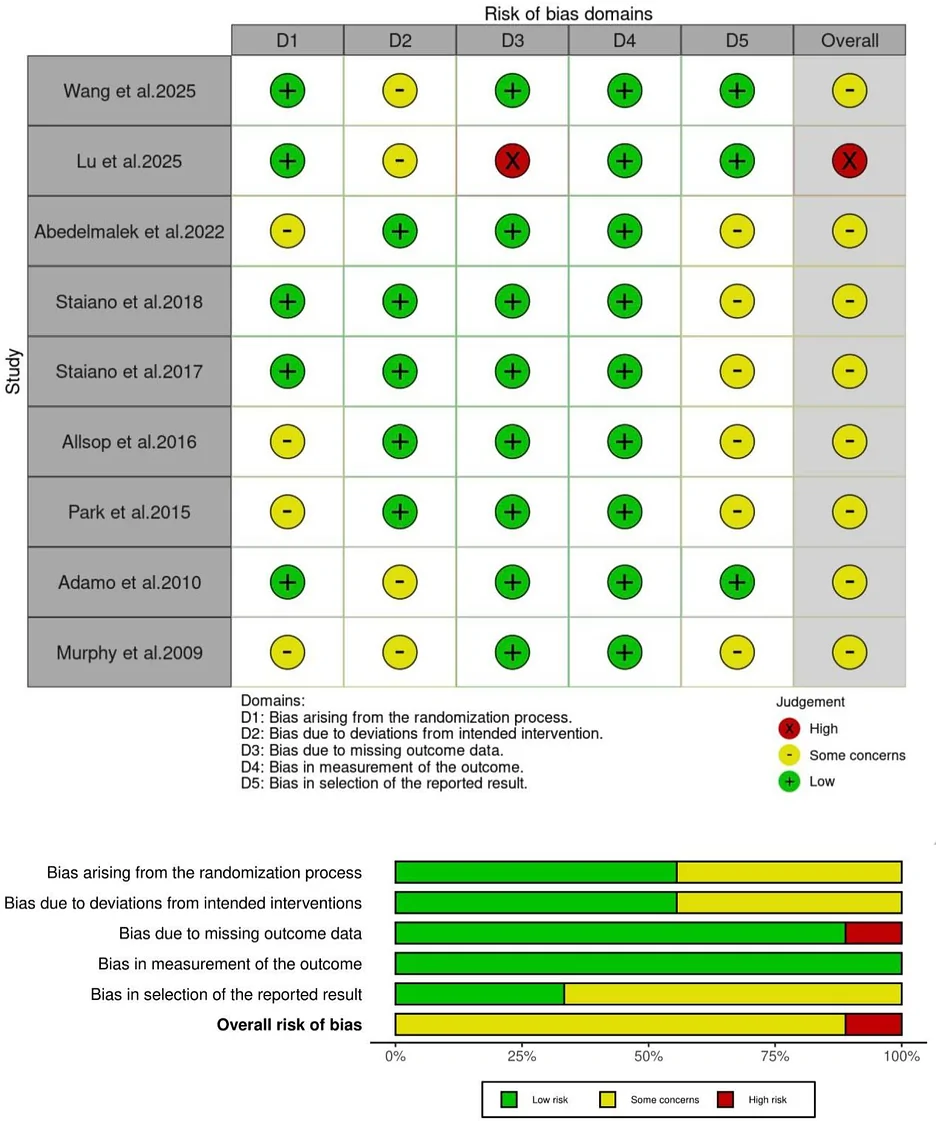
Risk of bias summary for the 9 randomized controlled trials assessed using RoB 2 [54-60,62,63].

Most included studies were judged as having some concerns regarding risk of bias. Of the 9 randomized trials [54-60,62,63] assessed using RoB 2, 8 (89%) were rated as having some concerns and 1 (11%) as high risk of bias. The most common issues were insufficient reporting of the randomization process (domain 1; 4/9 trials) and concerns regarding selection of the reported result because of the absence of clearly prespecified analysis plans or trial protocols (domain 5; 6/9 trials). The high-risk judgment was primarily related to missing outcome data (domain 3) in 1 trial. In contrast, bias arising from deviations from intended interventions (domain 2) and measurement of the outcome (domain 4) was generally judged to be low risk across the randomized trials (Figure 8).

The nonrandomized controlled study, assessed using ROBINS-I, was judged to be at serious overall risk of bias, mainly because of confounding, including nonconcurrent comparison and baseline imbalances. Additional concerns were identified for participant selection, deviations from intended interventions, missing data, and selection of the reported result (Multimedia Appendix 4).

The mean PEDro score across the 9 randomized trials was 6.3, indicating overall moderate methodological quality [35] (Multimedia Appendix 5).

### Reporting Bias and Sensitivity Analyses

Because all outcome-specific meta-analyses included fewer than 10 studies, no formal statistical assessment of reporting bias was performed. Egger regression test was not performed, and funnel plot asymmetry was not formally assessed because these methods are underpowered and potentially misleading when fewer than 10 studies are included [32,48-51].

Leave-one-out sensitivity analyses were conducted for glucose, TC, and triglycerides using the final dataset in which multiple intervention arms from the same trial were combined. For glucose, sequential removal of individual studies produced only small changes in the pooled estimate, with Hedges *g* ranging from −0.25 to −0.02; all estimates remained nonsignificant and were consistent with the main analysis (Hedges *g*=−0.16; 95% CI −0.57 to 0.24; *P*=.381; *I*²=65%). For TC, the pooled estimates ranged from Hedges *g*=−0.58 to −0.20 after omitting each study in turn, and all 95% CIs crossed the null. For triglycerides, the pooled estimates ranged from Hedges *g*=−0.22 to −0.07, with all results remaining nonsignificant. Overall, no single study disproportionately influenced the pooled estimates for glucose, TC, or triglycerides, although heterogeneity remained considerable for TC (Multimedia Appendix 6).

### Grade

Table 2 presents the official GRADEpro GDT summary of findings table. For each outcome, the table reports the standardized mean difference with its 95% CI, the number and design of contributing studies, the overall GRADE certainty rating, and concise reasons for downgrading. Certainty was very low for glucose and TC and low for insulin, triglycerides, HDL-C, and LDL-C. Publication bias was not assessed because fewer than 10 studies contributed to each outcome and did not result in an additional downgrade.

**Table 2.** GRADEpro GDT summary of findings: Exergaming compared with Nonexergaming controls for glycemic and lipid outcomes in children and adolescents^a^.

Outcomes	Anticipated absolute effects^b^ (95% CI)		Relative effect (95% CI)	No. of participants (studies)	Certainty of the evidence^c^ (GRADE^d^)^e^	Comments
	Risk with nonexergaming controls	Risk with exergaming				
Glucose assessed with: SMD^f^ (Hedges *g*)	—^g^	0.16 lower (0.57 lower to 0.24 higher)	—	528 (8 RCTs^h^ and 1 NRS)^i^	⊕○○○ Very low	PI^j^: −1.12 to 0.79. Downgraded for serious risk of bias, substantial inconsistency (*I*²=65%), and imprecision; the CI and PI crossed the null.
Insulin assessed with: SMD (Hedges *g*)	—	0.06 lower (0.35 lower to 0.23 higher)	—	308 (5 RCTs)	⊕⊕○○ Low	PI: −0.39 to 0.27. Downgraded for serious risk of bias and imprecision; the estimate was close to the null and the PI crossed the null.
TC assessed with: SMD (Hedges *g*)	—	0.39 lower (1.11 lower to 0.33 higher)	—	510 (8 RCTs and 1 NRS)	⊕○○○ Very low	PI: −1.70 to 0.93. Downgraded for serious risk of bias, considerable inconsistency (*I*²=77%), and imprecision; the CI and PI crossed the null.
Triglycerides assessed with: SMD (Hedges *g*)	—	0.17 lower (0.50 lower to 0.17 higher)	—	510 (8 RCTs and 1 NRS)	⊕⊕○○ Low	PI: −0.88 to 0.55. Downgraded for serious risk of bias and imprecision; the CI and PI crossed the null.
HDL-C^k^ assessed with: SMD (Hedges *g*)	—	0.14 lower (0.34 lower to 0.06 higher)	—	510 (8 RCTs and 1 NRS)	⊕⊕○○ Low	PI: −0.35 to 0.07. Downgraded for serious risk of bias and imprecision. Effect-size signs were reversed so negative values favor exergaming; original group means and SDs were unchanged. The CI and PI crossed the null.
LDL-C^l^ assessed with: SMD (Hedges *g*)	—	0.29 lower (0.55 lower to 0.04 lower)	—	510 (8 RCTs and 1 NRS)	⊕⊕○○ Low	PI: −0.73 to 0.14. Downgraded for serious risk of bias and imprecision; the pooled average favored exergaming, but the PI crossed the null.

a Patient or population: children and adolescents aged 18 years or younger from generally healthy or overweight and obese populations. Setting: home, school, laboratory, and community settings. Intervention: exergaming or active video game-based exercise. Comparison: nonexergaming controls, including usual care, no intervention, wait-list, seated rest, sedentary gaming, or conventional exercise. Follow-up: a single acute session to 24 weeks.

b For continuous outcomes summarized using SMDs, effects are expressed in SD units; therefore, baseline risks and relative effects are not reported. Negative SMDs favor exergaming. For HDL-C, effect-size signs were reversed because higher HDL-C is clinically favorable; original group means and SDs were unchanged.

c GRADE Working group grades of evidence- high certainty: we are very confident that the true effect lies close to that of the estimate of the effect; moderate certainty: we are moderately confident in the effect estimate; the true effect is likely to be close to the estimate, but it may be substantially different; low certainty: our confidence in the effect estimate is limited; the true effect may be substantially different from the estimate; very low certainty: we have very little confidence in the effect estimate; the true effect is likely to be substantially different from the estimate.

d GRADE: Grading of Recommendations Assessment, Development and Evaluation.

e GRADE certainty ratings: ⊕⊕⊕⊕, high certainty; ⊕⊕⊕○, moderate certainty; ⊕⊕○○, low certainty; and ⊕○○○, very low certainty.

f SMD: standardized mean difference.

g Not applicable.

h RCT: randomized controlled trial.

i NRS: nonrandomized study.

j PI: prediction interval.

k HDL-C: high-density lipoprotein cholesterol.

l LDL-C: low-density lipoprotein cholesterol.

## Discussion

### Principal Findings

This review synthesized the available intervention evidence on the effects of exergaming on glucose- and lipid-related metabolic outcomes in children and adolescents. Overall, the pooled analyses showed a small average reduction in LDL-C, whereas no significant pooled effects were observed for glucose, insulin, TC, triglycerides, or HDL-C. However, GRADE certainty was very low for glucose and TC and low for the other outcomes, and the 95% PI for LDL-C crossed the null. The current evidence therefore suggests a limited and uncertain outcome-specific signal rather than consistent improvement across glucose and lipid metabolism.

The CIs and PIs should be interpreted together. Although the pooled Hedges *g* estimates suggested a small favorable average reduction in LDL-C and no statistically significant average effects for the remaining outcomes, the PIs were wider than the corresponding CIs and crossed the null for all outcomes. This indicates that the effect observed in a comparable future study may be smaller, absent, or potentially opposite in direction. This uncertainty is consistent with the observed heterogeneity, which was substantial for glucose, considerable for TC, moderate for triglycerides, and low for insulin, HDL-C, and LDL-C, and may reflect differences in participant characteristics, exergaming modality, intervention content and intensity, comparator conditions, outcome measures, intervention duration, and timing of outcome assessment. Interpretation is further tempered by risk-of-bias concerns: 8 [54-58,60,62,63] of 9 randomized trials had some concerns, 1 [59] was at high risk, and the nonrandomized study was at serious overall risk of bias because of confounding and baseline imbalances. Common concerns involved incomplete reporting of randomization, absence of prespecified analysis plans, missing outcome data, and selective reporting. Consistent with these limitations, GRADE certainty was very low for glucose and TC and low for insulin, triglycerides, HDL-C, and LDL-C, mainly because of risk of bias, inconsistency where present, and imprecision. Therefore, the pooled findings should be considered suggestive rather than definitive; any metabolic benefit remains uncertain in magnitude and generalizability [41].

Heterogeneity was substantial for glucose and considerable for TC, suggesting that study-level differences may have influenced the pooled estimates. The included studies differed in exergaming modality, comparator condition, intervention duration, session frequency, movement demands, and participant characteristics, including age, sex, and weight status. Exploratory subgroup analyses suggested potential between-subgroup differences for glucose by sex composition and intervention type, for TC by sex composition, for triglycerides by sex composition and exergaming mode, and for HDL-C by age group. However, these findings should be interpreted with caution because several subgroups contained only one or two studies, and some apparent subgroup effects were driven by single-study estimates. Thus, the subgroup findings should be viewed as hypothesis-generating rather than confirmatory.

Taken together, these findings suggest that exergaming may be better positioned as a complementary strategy for increasing physical activity engagement in children and adolescents rather than as a stand-alone intervention for broad metabolic improvement. Its practical value may lie in replacing part of sedentary screen time with physically active gameplay and improving participation through interactive, enjoyable, and feedback-based formats [13-17]. However, the current evidence is not sufficient to support exergaming as a substitute for structured exercise training when the primary goal is to improve glucose and lipid metabolism. Future trials should determine whether more standardized, adequately intense, and longer-duration exergaming programs can produce clinically meaningful metabolic benefits, particularly in children and adolescents with elevated baseline metabolic risk.

### Intervention Effects

In this review, exergaming was associated with a small but statistically significant average reduction in LDL-C, with low between-study heterogeneity. LDL-C appeared to show a clearer pooled average signal than glucose, insulin, TC, triglycerides, or HDL-C in the current dataset. However, because the certainty of evidence was low and the 95% PI crossed the null, this result should not be interpreted as definitive evidence that exergaming consistently lowers LDL-C in all settings. The current finding is best understood as a promising but still uncertain average signal.

This result is only partly consistent with previous reviews. A recent review of exergaming in children and adolescents with overweight or obesity did not find a significant overall effect on LDL-C, although some favorable cardiometabolic or subgroup signals were reported [23]. Another review reported improvements in TC among adolescents with overweight or obesity [64]. Differences between reviews may be explained by variation in inclusion criteria, newly available trials, participant characteristics, baseline metabolic status, comparator conditions, and analytic methods. In particular, the present review included both acute and chronic interventions and considered different exergaming modalities, including commercial, customized, and VR-based programs. Because lipid responses to exercise are influenced by intervention dose and modality, differences in platform, movement demands, intervention length, weekly frequency, and intensity control may materially affect whether LDL-C changes are detectable. Evidence from pediatric exercise studies also suggests that lipid improvements are more likely when exercise provides sufficient duration, frequency, and aerobic stimulus, rather than when physical activity exposure is brief or weakly structured [65].

The most plausible explanation is not that exergaming itself has a direct LDL-C-lowering effect, but that some exergaming programs may deliver enough repeated active movement to shift energy expenditure and lipid handling. LDL-C may be more likely than glucose or insulin to show a detectable change in this context because it can reflect cumulative adaptations to repeated physical activity exposure over several weeks, whereas glucose and insulin are strongly influenced by fasting status, pubertal insulin sensitivity, baseline metabolic risk, and short-term dietary variation. Mechanistically, sufficiently active exergaming may replace sedentary screen time with whole-body movement, increase skeletal muscle energy use, and improve lipoprotein metabolism, including pathways related to triglyceride-rich lipoprotein clearance, LDL particle handling, reverse cholesterol transport, and exercise-induced changes in lipid transport [65-68]. Exercise training has been shown to affect lipid metabolism, but these effects are dose- and modality-dependent rather than automatic consequences of any activity exposure [65,68]. Thus, the LDL-C reduction observed in this review may reflect the effect of more physically demanding or higher-dose exergaming interventions, not a uniform effect of exergaming as a whole. Future trials should test whether LDL-C benefits require specific thresholds of moderate-to-vigorous intensity exposure, longer intervention duration, or elevated baseline dyslipidemia risk.

### Glucose and Other Lipid Outcomes

In this review, exergaming did not show significant pooled effects on glucose, insulin, TC, triglycerides, or HDL-C. This pattern is broadly consistent with previous reviews suggesting that exergaming may improve some health-related outcomes, such as body composition, physical fitness, or blood pressure, while its effects on glucose regulation and most lipid components remain inconsistent or nonsignificant [23]. Therefore, these findings should not be interpreted simply as evidence of no value, but rather as evidence that current exergaming trials have not yet demonstrated consistent metabolic benefits beyond the LDL-C signal.

One likely explanation is the limited room for measurable improvement in many included participants. Although most studies enrolled children or adolescents with overweight or obesity, excess body weight does not necessarily indicate abnormal baseline glucose or lipid levels. Children and adolescents with obesity may maintain normal fasting glucose for a considerable period through compensatory insulin secretion, and puberty-related reductions in insulin sensitivity may further increase biological variability in glucose and insulin outcomes [69-72]. Similarly, dyslipidemia is not present in all pediatric populations with obesity, and baseline TC, triglycerides, or HDL-C values may remain close to normal in some participants [73]. These factors may reduce the likelihood of detecting short- to mid-term intervention effects.

Another explanation is the heterogeneity of exergaming prescriptions. Compared with structured exercise training, exergaming interventions are often less standardized in intensity, progression, total exercise volume, and movement demands [13,14,19]. Differences in game type, platform, supervision, intervention duration, and comparator condition may dilute pooled effects, especially for outcomes that are sensitive to diet, recent activity, pubertal status, and baseline metabolic risk. Nevertheless, these nonsignificant results are still informative: they suggest that exergaming should currently be positioned primarily as a strategy to increase physical activity engagement and reduce sedentary screen time, rather than as a guaranteed intervention for improving all glucose and lipid biomarkers. Future studies should target participants with higher baseline metabolic risk and use better-standardized prescriptions with objective monitoring of exercise intensity and total activity dose.

### Implications for Future Research

Future research should focus on clarifying the conditions under which exergaming can produce clinically meaningful metabolic benefits. First, trials should standardize and clearly report intervention prescriptions, including frequency, session duration, intervention length, intensity, progression, and supervision. Because current evidence suggests limited and outcome-specific metabolic effects, future studies should objectively verify the delivered exercise dose using heart rate, accelerometry, or other wearable measures rather than assuming that all exergaming provides sufficient moderate-to-vigorous physical activity. This is important because physical activity guidelines for children and adolescents emphasize regular moderate-to-vigorous activity, and exergaming intensity can vary substantially by game format and movement demand [7,74]. Second, future interventions should optimize game content and device features to provide a more controllable training stimulus. Platforms with whole-body engagement, adjustable difficulty, real-time feedback, and progressive workload may be more likely to achieve sufficient cumulative activity exposure than low-intensity or intermittent games. Recent studies also suggest that some VR-based active games can elicit meaningful energy expenditure and maintain enjoyment, but this depends on the specific game and intensity profile rather than VR use alone [75,76]. Third, future trials should stratify or target participants according to baseline metabolic risk, including dyslipidemia, insulin resistance, obesity severity, and pubertal stage, because participants with normal baseline biomarkers may have limited room for improvement. Finally, larger preregistered trials with rigorous randomization, allocation concealment, longer follow-up, and better control or documentation of diet, habitual physical activity, sleep, and other cointerventions are needed. Incorporating more sensitive metabolic outcomes, such as insulin resistance indices, lipoprotein subfractions, or continuous glucose-related measures, may also help determine whether exergaming has effects that are not captured by conventional fasting biomarkers alone.

### Strengths and Limitations

This study has several strengths. First, the review was conducted in accordance with the PRISMA 2020 guideline and was prospectively registered. Second, we conducted a comprehensive literature search across 6 core databases, including CENTRAL, and incorporated backward and forward citation searching to provide a more current and transparent synthesis of evidence on exergaming and glucose- and lipid-related outcomes in children and adolescents. Third, we used a conservative analytical approach by applying Hartung-Knapp-Sidik-Jonkman-adjusted random-effects models, correcting for double counting in multiarm trials, orienting effect estimates consistently across outcomes, and reporting PIs where appropriate. Fourth, we explored potential sources of heterogeneity using subgroup analyses and exploratory meta-regression, assessed risk of bias using RoB 2 and ROBINS-I, summarized methodological quality using the PEDro scale, and evaluated the certainty of evidence using the GRADE approach. Sensitivity analyses were also performed to examine the stability of the pooled estimates.

Several limitations should also be acknowledged. First, the number of included studies was limited, and many trials had relatively small sample sizes, which reduced statistical power, including power for subgroup and meta-regression analyses [77]. Second, intervention protocols differed substantially in exergaming modality, intensity, frequency, duration, supervision, and comparator conditions, and heterogeneity was substantial or considerable for some outcomes. Third, many studies provided insufficient information on allocation concealment, preregistration, or prespecified analysis plans, contributing to the predominance of “some concerns’” judgments in the risk-of-bias assessment. Fourth, the included studies often provided limited information on baseline metabolic risk, pubertal status, diet, habitual physical activity, and intervention intensity, which restricted our ability to explain why some outcomes responded while others did not. Finally, the certainty of evidence ranged from very low to low across outcomes; therefore, the current findings should be interpreted cautiously and require confirmation in larger, preregistered, and more rigorously designed trials.

### Conclusion

This review extends previous exergaming reviews by focusing specifically on glycemic and lipid biomarkers in children and adolescents and by examining potential moderators of metabolic responses. Whereas earlier reviews mainly emphasized physical activity, adiposity, fitness, or psychosocial outcomes, this study provides a targeted synthesis of cardiometabolic biomarkers and highlights LDL-C as the most consistent but still uncertain lipid outcome. From a practical perspective, exergaming may offer an engaging and scalable option for schools, families, and community settings to complement conventional physical activity strategies. Nevertheless, the findings should be interpreted cautiously because the certainty of evidence ranged from very low to low, several studies had risk-of-bias concerns, intervention protocols were heterogeneous, and PIs crossed the null. Future adequately powered, preregistered randomized trials with standardized metabolic outcomes and clearly reported intervention dose are needed before firm metabolic conclusions can be drawn.

## Supplementary material

10.2196/93610 Multimedia Appendix 1 Full search strategies.

10.2196/93610 Multimedia Appendix 2 Subgroup outputs.

10.2196/93610 Multimedia Appendix 3 Meta-regression Outputs.

10.2196/93610 Multimedia Appendix 4 ROBINS-I Assessment.

10.2196/93610 Multimedia Appendix 5 PEDro.

10.2196/93610 Multimedia Appendix 6 Sensitivity analysis.

10.2196/93610 Checklist 1 PRISMA checklist.

## Abbreviations

GDT: Guideline Development Tool
GRADE: Grading of Recommendations Assessment, Development and Evaluation
HDL-C: high-density lipoprotein cholesterol
LDL-C: low-density lipoprotein cholesterol
PEDro: Physiotherapy Evidence Database
PI: prediction interval
PICOS: Population, Intervention, Comparison, Outcomes, and Study design
PRISMA: Preferred Reporting Items for Systematic Reviews and Meta-Analyses
RoB 2: Cochrane Risk of Bias 2
ROBINS-I: Risk Of Bias In Non-randomized Studies of Interventions
TC: total cholesterol
VR: virtual reality
