# Supplementary material for: Effects and Moderators of Exergaming on Glucose and Lipid Metabolism in Children and Adolescents: Systematic Review and Meta-Analysis
Source: JMIR Serious Games. 2026 Sep 15;14:e93610. doi: 10.2196/93610 (PMC13579656; doi:10.2196/93610)
Supplement: Supplementary file 1 [file games-v14-e93610-s001.docx]

**Full Search Strategies**

Coverage: database inception through April 27, 2026. The six database searches retrieved 10,894 records before deduplication. The strategies combined exergaming and pediatric-population concepts; outcome terms were not required.

| **Database** | **Complete search strategy** | **Records retrieved** |
| --- | --- | --- |
| **PubMed** | (exergam*[tiab] OR exergame*[tiab] OR "active video gam*"[tiab] OR "active videogam*"[tiab] OR "interactive video gam*"[tiab] OR "exercise video gam*"[tiab] OR "game-based exercise"[tiab] OR "game based exercise"[tiab] OR "game-based physical activ*"[tiab] OR "active gaming"[tiab] OR "active videogaming"[tiab] OR "active video gaming"[tiab] OR "Wii Fit"[tiab] OR "Dance Dance Revolution"[tiab] OR "GameBike"[tiab] OR ((Wii[tiab] OR Kinect[tiab] OR Xbox[tiab] OR "virtual reality"[tiab] OR VR[tiab]) AND (exercise[tiab] OR training[tiab] OR active[tiab] OR physical[tiab]))) AND ("Child"[Mesh] OR "Adolescent"[Mesh] OR child*[tiab] OR adolescen*[tiab] OR teen*[tiab] OR youth*[tiab] OR pediatric*[tiab] OR paediatric*[tiab] OR schoolchild*[tiab] OR "school-age*"[tiab] OR "school age*"[tiab]) | 2,139 |
| **Embase** | (exergam*:ti,ab,kw OR exergame*:ti,ab,kw OR 'active video gam*':ti,ab,kw OR 'active videogam*':ti,ab,kw OR 'interactive video gam*':ti,ab,kw OR 'exercise video gam*':ti,ab,kw OR 'game-based exercise':ti,ab,kw OR 'game based exercise':ti,ab,kw OR 'game-based physical activ*':ti,ab,kw OR 'active gaming':ti,ab,kw OR 'active video gaming':ti,ab,kw OR 'active videogaming':ti,ab,kw OR 'wii fit':ti,ab,kw OR 'dance dance revolution':ti,ab,kw OR gamebike:ti,ab,kw OR ((wii:ti,ab,kw OR kinect:ti,ab,kw OR xbox:ti,ab,kw OR 'virtual reality':ti,ab,kw OR vr:ti,ab,kw) AND (exercise:ti,ab,kw OR training:ti,ab,kw OR active:ti,ab,kw OR physical:ti,ab,kw))) AND ('child'/exp OR 'adolescent'/exp OR child*:ti,ab,kw OR adolescen*:ti,ab,kw OR teen*:ti,ab,kw OR youth*:ti,ab,kw OR pediatric*:ti,ab,kw OR paediatric*:ti,ab,kw OR schoolchild*:ti,ab,kw OR 'school-age*':ti,ab,kw OR 'school age*':ti,ab,kw) | 2,955 |
| **Scopus** | TITLE-ABS-KEY(exergam* OR exergame* OR "active video game" OR "active video games" OR "active videogame" OR "active videogames" OR "interactive video game" OR "interactive video games" OR "exercise video game" OR "exercise video games" OR "game-based exercise" OR "game based exercise" OR "game-based physical activity" OR "game based physical activity" OR "active gaming" OR "active video gaming" OR "active videogaming" OR "Wii Fit" OR "Dance Dance Revolution" OR GameBike OR ((Wii OR Kinect OR Xbox OR "virtual reality" OR VR) AND (exercise OR training OR active OR physical))) AND TITLE-ABS-KEY(child* OR adolescen* OR teen* OR youth* OR pediatric* OR paediatric* OR schoolchild* OR "school-age" OR "school age") | 1,312 |
| **Web of Science** | TS=(exergam* OR exergame* OR "active video game" OR "active video games" OR "active videogame" OR "active videogames" OR "interactive video game" OR "interactive video games" OR "exercise video game" OR "exercise video games" OR "game-based exercise" OR "game based exercise" OR "game-based physical activity" OR "game based physical activity" OR "active gaming" OR "active video gaming" OR "active videogaming" OR "Wii Fit" OR "Dance Dance Revolution" OR GameBike OR ((Wii OR Kinect OR Xbox OR "virtual reality" OR VR) AND (exercise OR training OR active OR physical))) AND TS=(child* OR adolescen* OR teen* OR youth* OR pediatric* OR paediatric* OR schoolchild* OR "school-age" OR "school age") | 1,427 |
| **Psychology and Behavioral Sciences Collection (EBSCOhost)** | TX (exergam* OR exergame* OR "active video game" OR "active video games" OR "active videogame" OR "active videogames" OR "interactive video game" OR "interactive video games" OR "exercise video game" OR "exercise video games" OR "game-based exercise" OR "game based exercise" OR "game-based physical activity" OR "game based physical activity" OR "active gaming" OR "active video gaming" OR "active videogaming" OR "Wii Fit" OR "Dance Dance Revolution" OR GameBike OR ((Wii OR Kinect OR Xbox OR "virtual reality" OR VR) AND (exercise OR training OR active OR physical))) AND TX (child* OR adolescen* OR teen* OR youth* OR pediatric* OR paediatric* OR schoolchild* OR "school-age" OR "school age") | 1,497 |
| **CENTRAL** | (exergam* OR exergame* OR "active video game" OR "active video games" OR "active videogame" OR "active videogames" OR "interactive video game" OR "interactive video games" OR "exercise video game" OR "exercise video games" OR "game-based exercise" OR "game based exercise" OR "game-based physical activity" OR "game based physical activity" OR "active gaming" OR "active video gaming" OR "active videogaming" OR "Wii Fit" OR "Dance Dance Revolution" OR GameBike OR ((Wii OR Kinect OR Xbox OR "virtual reality" OR VR) AND (exercise OR training OR active OR physical))) AND (child* OR adolescen* OR teen* OR youth* OR pediatric* OR paediatric* OR schoolchild* OR "school-age" OR "school age") | 1,564 |

Abbreviations: CENTRAL, Cochrane Central Register of Controlled Trials; EBSCOhost, EBSCO Information Services platform.
