# Supplementary material for: Effects and Moderators of Exergaming on Glucose and Lipid Metabolism in Children and Adolescents: Systematic Review and Meta-Analysis
Source: JMIR Serious Games. 2026 Sep 15;14:e93610. doi: 10.2196/93610 (PMC13579656; doi:10.2196/93610)
Supplement: Supplementary file 2 [file games-v14-e93610-s002.docx]

**Subgroup Analysis Outputs**

**Subgroup analyses of Glucose**

| **Subgroup** | **Studies (participants)** | **Hedges g** | **95% CI** | **P (within)** | **Q** | **I²** | **P (between)** |
| --- | --- | --- | --- | --- | --- | --- | --- |
| **Age** |  |  |  |  |  |  | **.273** |
| Children | 4 (201) | -0.39 | [-1.36, 0.58] | .290 | 10.98 | 73% |  |
| Adolescents | 5 (327) | 0.01 | [-0.53, 0.54] | .980 | 8.39 | 52% |  |
| **Sex** |  |  |  |  |  |  | **.001** |
| Mixed sex | 7 (448) | 0.02 | [-0.28, 0.31] | .900 | 8.16 | 26% |  |
| Male only | 1 (42) | -1.31 | [-1.98, -0.63] | <.001 | n/a | n/a |  |
| Female only | 1 (38) | -0.34 | [-0.99, 0.30] | .300 | n/a | n/a |  |
| **Weight status** |  |  |  |  |  |  | **.228** |
| Overweight/obese | 7 (377) | 0.01 | [-0.32, 0.35] | .930 | 8.49 | 29% |  |
| Normal-weight | 2 (151) | -0.70 | [-7.98, 6.59] | .440 | 8.53 | 88% |  |
| **Exergaming mode** |  |  |  |  |  |  | **.070** |
| Commercial | 6 (202) | -0.30 | [-1.00, 0.39] | .310 | 14.95 | 67% |  |
| Customized | 2 (188) | -0.11 | [-0.87, 0.65] | .320 | 0.16 | 0% |  |
| VR-based | 1 (138) | 0.30 | [-0.06, 0.65] | .100 | n/a | n/a |  |
| **Intervention type** |  |  |  |  |  |  | **.037** |
| Chronic | 7 (462) | 0.01 | [-0.27, 0.30] | .930 | 8.09 | 26% |  |
| Acute | 2 (66) | -0.91 | [-6.32, 4.50] | .278 | 2.52 | 60% |  |

Abbreviations: CI, confidence interval; I², proportion of variability attributable to heterogeneity; n/a, not applicable; P (within), test of the pooled effect within a subgroup; P (between), test of differences between subgroups; Q, Cochran Q statistic. Except for HDL-C (see the HDL-C page), negative Hedges g values favor exergaming. These analyses are exploratory; single-study subgroups cannot provide within-subgroup heterogeneity estimates.

**Subgroup analyses of Insulin**

| **Subgroup** | **Studies (participants)** | **Hedges g** | **95% CI** | **P (within)** | **Q** | **I²** | **P (between)** |
| --- | --- | --- | --- | --- | --- | --- | --- |
| **Age** |  |  |  |  |  |  | **.475** |
| Adolescents | 3 (194) | 0.00 | [-0.52, 0.52] | .989 | 1.32 | 0% |  |
| Children | 2 (114) | -0.19 | [-3.11, 2.74] | .565 | 1.25 | 20% |  |
| **Exergaming mode** |  |  |  |  |  |  | **.809** |
| VR-based | 1 (138) | 0.00 | [-0.35, 0.35] | 1.000 | n/a | n/a |  |
| Commercial | 3 (91) | -0.19 | [-1.23, 0.86] | .519 | 2.51 | 20% |  |
| Customized | 1 (79) | -0.02 | [-0.49, 0.45] | .930 | n/a | n/a |  |

Abbreviations: CI, confidence interval; I², proportion of variability attributable to heterogeneity; n/a, not applicable; P (within), test of the pooled effect within a subgroup; P (between), test of differences between subgroups; Q, Cochran Q statistic. Except for HDL-C (see the HDL-C page), negative Hedges g values favor exergaming. These analyses are exploratory; single-study subgroups cannot provide within-subgroup heterogeneity estimates.

**Subgroup analyses of HDL-C**

HDL-C values are shown in the conventional direction in these original subgroup outputs, so positive values favor higher HDL-C with exergaming. The main pooled forest plot reverse-oriented HDL-C to make negative values consistently favor exergaming; the sign transformation does not change P values or subgroup tests.

| **Subgroup** | **Studies (participants)** | **Hedges g** | **95% CI** | **P (within)** | **Q** | **I²** | **P (between)** |
| --- | --- | --- | --- | --- | --- | --- | --- |
| **Age** |  |  |  |  |  |  | **.001** |
| Adolescents | 6 (351) | 0.24 | [-0.00, 0.49] | .052 | 3.86 | 0% |  |
| Children | 3 (159) | -0.10 | [-0.29, 0.08] | .135 | 0.13 | 0% |  |
| **Sex** |  |  |  |  |  |  | **.436** |
| Mixed sex | 7 (448) | 0.13 | [-0.10, 0.36] | .218 | 5.40 | 0% |  |
| Male only | 1 (24) | -0.32 | [-1.13, 0.48] | .400 | n/a | n/a |  |
| Female only | 1 (38) | 0.35 | [-0.29, 0.99] | .300 | n/a | n/a |  |
| **Weight status** |  |  |  |  |  |  | **.062** |
| Overweight/obese | 8 (401) | 0.05 | [-0.13, 0.24] | .531 | 3.94 | 0% |  |
| Normal-weight | 1 (109) | 0.44 | [0.06, 0.82] | .020 | n/a | n/a |  |
| **Exergaming mode** |  |  |  |  |  |  | **.157** |
| VR-based | 1 (138) | 0.25 | [-0.11, 0.60] | .180 | n/a | n/a |  |
| Customized | 2 (103) | -0.12 | [-1.64, 1.41] | .513 | 0.34 | 0% |  |
| Commercial | 6 (269) | 0.15 | [-0.16, 0.45] | .279 | 4.78 | 0% |  |
| **Intervention type** |  |  |  |  |  |  | **.689** |
| Chronic | 8 (486) | 0.13 | [-0.09, 0.36] | .204 | 6.89 | 0% |  |
| Acute | 1 (24) | -0.04 | [-0.84, 0.77] | .970 | n/a | n/a |  |

Abbreviations: CI, confidence interval; I², proportion of variability attributable to heterogeneity; n/a, not applicable; P (within), test of the pooled effect within a subgroup; P (between), test of differences between subgroups; Q, Cochran Q statistic. Except for HDL-C (see the HDL-C page), negative Hedges g values favor exergaming. These analyses are exploratory; single-study subgroups cannot provide within-subgroup heterogeneity estimates.

**Subgroup analyses of LDL-C**

| **Subgroup** | **Studies (participants)** | **Hedges g** | **95% CI** | **P (within)** | **Q** | **I²** | **P (between)** |
| --- | --- | --- | --- | --- | --- | --- | --- |
| **Age** |  |  |  |  |  |  | **.931** |
| Adolescents | 6 (351) | -0.32 | [-0.63, -0.01] | .046 | 5.66 | 12% |  |
| Children | 3 (159) | -0.30 | [-1.40, 0.81] | .369 | 4.53 | 56% |  |
| **Sex** |  |  |  |  |  |  | **.179** |
| Mixed sex | 7 (448) | -0.34 | [-0.60, -0.07] | .020 | 6.80 | 12% |  |
| Male only | 1 (24) | -0.62 | [-1.45, 0.20] | .200 | n/a | n/a |  |
| Female only | 1 (38) | 0.24 | [-0.40, 0.88] | .530 | n/a | n/a |  |
| **Weight status** |  |  |  |  |  |  | **.397** |
| Overweight/obese | 8 (401) | -0.25 | [-0.56, 0.05] | .092 | 9.67 | 28% |  |
| Normal-weight | 1 (109) | -0.45 | [-0.83, -0.07] | .020 | n/a | n/a |  |
| **Exergaming mode** |  |  |  |  |  |  | **.632** |
| VR-based | 1 (138) | -0.46 | [-0.82, -0.11] | .010 | n/a | n/a |  |
| Customized | 2 (103) | -0.22 | [-3.92, 3.47] | .586 | 1.61 | 38% |  |
| Commercial | 6 (269) | -0.25 | [-0.66, 0.16] | .177 | 7.46 | 33% |  |
| **Intervention type** |  |  |  |  |  |  | **.325** |
| Chronic | 8 (486) | -0.32 | [-0.59, -0.05] | .027 | 9.25 | 24% |  |
| Acute | 1 (24) | 0.10 | [-0.70, 0.90] | .880 | n/a | n/a |  |

Abbreviations: CI, confidence interval; I², proportion of variability attributable to heterogeneity; n/a, not applicable; P (within), test of the pooled effect within a subgroup; P (between), test of differences between subgroups; Q, Cochran Q statistic. Except for HDL-C (see the HDL-C page), negative Hedges g values favor exergaming. These analyses are exploratory; single-study subgroups cannot provide within-subgroup heterogeneity estimates.

**Subgroup analyses of Total cholesterol**

| **Subgroup** | **Studies (participants)** | **Hedges g** | **95% CI** | **P (within)** | **Q** | **I²** | **P (between)** |
| --- | --- | --- | --- | --- | --- | --- | --- |
| **Age** |  |  |  |  |  |  | **.640** |
| Adolescents | 6 (351) | -0.60 | [-2.09, 0.88] | .344 | 30.19 | 83% |  |
| Children | 3 (159) | -0.31 | [-1.39, 0.77] | .345 | 4.39 | 54% |  |
| **Sex** |  |  |  |  |  |  | **<.001** |
| Mixed sex | 7 (448) | -0.28 | [-0.54, -0.03] | .034 | 6.35 | 6% |  |
| Male only | 1 (24) | -3.73 | [-5.14, -2.33] | <.001 | n/a | n/a |  |
| Female only | 1 (38) | 0.46 | [-0.19, 1.11] | .165 | n/a | n/a |  |
| **Weight status** |  |  |  |  |  |  | **.409** |
| Overweight/obese | 8 (401) | -0.52 | [-1.50, 0.45] | .246 | 33.91 | 79% |  |
| Normal-weight | 1 (109) | -0.15 | [-0.52, 0.23] | .438 | n/a | n/a |  |
| **Exergaming mode** |  |  |  |  |  |  | **.328** |
| VR-based | 1 (138) | -0.48 | [-0.83, -0.12] | .008 | n/a | n/a |  |
| Customized | 2 (103) | -1.81 | [-25.46, 21.85] | .509 | 24.30 | 96% |  |
| Commercial | 6 (269) | -0.17 | [-0.60, 0.27] | .374 | 8.08 | 38% |  |
| **Intervention type** |  |  |  |  |  |  | **.420** |
| Chronic | 8 (486) | -0.52 | [-1.49, 0.44] | .239 | 34.27 | 80% |  |
| Acute | 1 (24) | -0.06 | [-0.86, 0.74] | .883 | n/a | n/a |  |

Abbreviations: CI, confidence interval; I², proportion of variability attributable to heterogeneity; n/a, not applicable; P (within), test of the pooled effect within a subgroup; P (between), test of differences between subgroups; Q, Cochran Q statistic. Except for HDL-C (see the HDL-C page), negative Hedges g values favor exergaming. These analyses are exploratory; single-study subgroups cannot provide within-subgroup heterogeneity estimates.

**Subgroup analyses of Triglycerides**

| **Subgroup** | **Studies (participants)** | **Hedges g** | **95% CI** | **P (within)** | **Q** | **I²** | **P (between)** |
| --- | --- | --- | --- | --- | --- | --- | --- |
| **Age** |  |  |  |  |  |  | **.429** |
| Adolescents | 6 (351) | -0.25 | [-0.87, 0.37] | .348 | 14.95 | 67% |  |
| Children | 3 (159) | -0.06 | [-0.22, 0.10] | .268 | 0.10 | 0% |  |
| **Sex** |  |  |  |  |  |  | **.005** |
| Mixed sex | 7 (448) | -0.13 | [-0.34, 0.08] | .182 | 5.13 | 0% |  |
| Male only | 1 (24) | -1.51 | [-2.44, -0.59] | <.001 | n/a | n/a |  |
| Female only | 1 (38) | 0.32 | [-0.32, 0.96] | .320 | n/a | n/a |  |
| **Weight status** |  |  |  |  |  |  | **.174** |
| Overweight/obese | 8 (401) | -0.22 | [-0.60, 0.16] | .211 | 12.95 | 46% |  |
| Normal-weight | 1 (109) | 0.12 | [-0.26, 0.50] | .535 | n/a | n/a |  |
| **Exergaming mode** |  |  |  |  |  |  | **.028** |
| VR-based | 1 (138) | -0.45 | [-0.81, -0.10] | .013 | n/a | n/a |  |
| Customized | 2 (103) | -0.71 | [-10.22, 8.80] | .517 | 8.01 | 88% |  |
| Commercial | 6 (269) | 0.04 | [-0.15, 0.23] | .602 | 1.75 | 0% |  |
| **Intervention type** |  |  |  |  |  |  | **.940** |
| Chronic | 8 (486) | -0.17 | [-0.56, 0.22] | .337 | 15.54 | 55% |  |
| Acute | 1 (24) | -0.14 | [-0.94, 0.67] | .800 | n/a | n/a |  |

Abbreviations: CI, confidence interval; I², proportion of variability attributable to heterogeneity; n/a, not applicable; P (within), test of the pooled effect within a subgroup; P (between), test of differences between subgroups; Q, Cochran Q statistic. Except for HDL-C (see the HDL-C page), negative Hedges g values favor exergaming. These analyses are exploratory; single-study subgroups cannot provide within-subgroup heterogeneity estimates.
