# Supplementary material for: Effects and Moderators of Exergaming on Glucose and Lipid Metabolism in Children and Adolescents: Systematic Review and Meta-Analysis
Source: JMIR Serious Games. 2026 Sep 15;14:e93610. doi: 10.2196/93610 (PMC13579656; doi:10.2196/93610)
Supplement: Supplementary file 3 [file games-v14-e93610-s003.docx]

**Meta-Regression Outputs**

Each panel shows an exploratory univariable random-effects meta-regression of Hedges g against total intervention duration (weeks), weekly session frequency, session duration (minutes), baseline BMI, and mean participant age. No moderator slope was statistically significant. For HDL-C only, the original plot uses the conventional positive direction; the main forest plot reverse-oriented HDL-C for consistent clinical interpretation.

**Glucose**

**Insulin**

**HDL-C**

**LDL-C**

**Total cholesterol**

**Triglycerides**
