# Supplementary material for: Effects and Moderators of Exergaming on Glucose and Lipid Metabolism in Children and Adolescents: Systematic Review and Meta-Analysis
Source: JMIR Serious Games. 2026 Sep 15;14:e93610. doi: 10.2196/93610 (PMC13579656; doi:10.2196/93610)
Supplement: Supplementary file 4 [file games-v14-e93610-s004.docx]

**ROBINS-I Assessment**

ROBINS-I judgments for the nonrandomized controlled study. Overall risk of bias was serious, driven primarily by confounding, including the nonconcurrent comparison and baseline imbalances. Additional concerns involved participant selection, deviations from intended interventions, missing data, and selection of the reported result.

Study: Chae S, Yeo J, Hwang J, Kang HS. Effects of school-based "we fit" weight control programme in adolescents. Nurs Open. 2022;9(1):721-732. doi:10.1002/nop2.1125.
