# Supplementary material for: Effects and Moderators of Exergaming on Glucose and Lipid Metabolism in Children and Adolescents: Systematic Review and Meta-Analysis
Source: JMIR Serious Games. 2026 Sep 15;14:e93610. doi: 10.2196/93610 (PMC13579656; doi:10.2196/93610)
Supplement: Supplementary file 5 [file games-v14-e93610-s005.docx]

**PEDro Assessment of Randomized Trials**

The mean PEDro score was 6.3/10 across the 9 randomized trials. Higher scores indicate better methodological quality. The scores were used descriptively and were not used to exclude studies, weight effect estimates, or determine GRADE certainty. Item 1 concerns external validity and is not included in the total score.

| **Study** | **D1** | **D2** | **D3** | **D4** | **D5** | **D6** | **D7** | **D8** | **D9** | **D10** | **D11** | **Total** |
| --- | --- | --- | --- | --- | --- | --- | --- | --- | --- | --- | --- | --- |
| Wang et al., 2025 | Y | 1 | 1 | 1 | 0 | 0 | 1 | 1 | 1 | 1 | 1 | 8 |
| Lu et al., 2025 | Y | 1 | 0 | 1 | 0 | 0 | 1 | 0 | 0 | 1 | 1 | 5 |
| Abedelmalek et al., 2022 | Y | 1 | 0 | 1 | 0 | 0 | 1 | 1 | 0 | 1 | 1 | 6 |
| Staiano et al., 2018 | Y | 1 | 1 | 1 | 0 | 0 | 1 | 1 | 1 | 1 | 1 | 8 |
| Staiano et al., 2017 | Y | 1 | 1 | 1 | 0 | 0 | 1 | 1 | 1 | 1 | 1 | 8 |
| Allsop et al., 2016 | Y | 1 | 0 | 1 | 0 | 0 | 0 | 1 | 0 | 1 | 1 | 5 |
| Park et al., 2015 | Y | 1 | 0 | 1 | 0 | 0 | 0 | 1 | 0 | 1 | 1 | 5 |
| Adamo et al., 2010 | Y | 1 | 1 | 1 | 0 | 0 | 0 | 1 | 0 | 1 | 1 | 6 |
| Murphy et al., 2009 | Y | 1 | 0 | 1 | 0 | 0 | 1 | 1 | 0 | 1 | 1 | 6 |

Coding: Y, eligibility criterion specified; 1, criterion satisfied; 0, criterion not satisfied.

**PEDro criteria**

| **Item** | **Criterion** |
| --- | --- |
| **1** | Eligibility criteria were specified (not included in the total score). |
| **2** | Participants were randomly allocated to groups; in a crossover study, participants were randomly allocated the order in which treatments were received. |
| **3** | Allocation was concealed. |
| **4** | Groups were similar at baseline regarding the most important prognostic indicators. |
| **5** | All participants were blinded. |
| **6** | All therapists who administered the therapy were blinded. |
| **7** | All assessors who measured at least one key outcome were blinded. |
| **8** | Measures of at least one key outcome were obtained from more than 85% of participants initially allocated to groups. |
| **9** | All participants with available outcome measures received the allocated treatment or control condition, or data for at least one key outcome were analyzed by intention to treat. |
| **10** | Between-group statistical comparisons were reported for at least one key outcome. |
| **11** | The study provided both point estimates and measures of variability for at least one key outcome. |
