# Supplementary material for: Effects and Moderators of Exergaming on Glucose and Lipid Metabolism in Children and Adolescents: Systematic Review and Meta-Analysis
Source: JMIR Serious Games. 2026 Sep 15;14:e93610. doi: 10.2196/93610 (PMC13579656; doi:10.2196/93610)
Supplement: Supplementary file 6 [file games-v14-e93610-s006.docx]

**Leave-One-Out Sensitivity Analyses**

**Glucose**

Hedges g ranged from -0.25 to -0.02; every 95% CI crossed the null. Each row reports the random-effects model after omitting the named study. Negative Hedges g values favor exergaming.

| **Omitted study** | **Hedges g** | **95% CI** | **P value** | **I²** |
| --- | --- | --- | --- | --- |
| Omitting Wang et al., 2025 | -0.25 | -0.69 to 0.20 | .233 | 57.2% |
| Omitting Chae et al., 2022 | -0.17 | -0.66 to 0.32 | .441 | 69.0% |
| Omitting Staiano et al., 2018 | -0.19 | -0.67 to 0.29 | .375 | 69.0% |
| Omitting Staiano et al., 2017 | -0.15 | -0.62 to 0.33 | .492 | 68.4% |
| Omitting Allsop et al., 2016 | -0.02 | -0.29 to 0.25 | .872 | 24.9% |
| Omitting Park et al., 2015 | -0.14 | -0.61 to 0.33 | .503 | 68.1% |
| Omitting Adamo et al., 2010 | -0.23 | -0.62 to 0.16 | .201 | 63.5% |
| Omitting Murphy et al., 2009 | -0.15 | -0.63 to 0.33 | .482 | 68.6% |
| Omitting Lu et al., 2025 | -0.19 | -0.67 to 0.30 | .392 | 69.1% |

Abbreviations: CI, confidence interval; I², proportion of variability attributable to heterogeneity.

**Total cholesterol**

Hedges g ranged from -0.58 to -0.20; every 95% CI crossed the null. Each row reports the random-effects model after omitting the named study. Negative Hedges g values favor exergaming.

| **Omitted study** | **Hedges g** | **95% CI** | **P value** | **I²** |
| --- | --- | --- | --- | --- |
| Omitting Wang et al., 2025 | -0.48 | -1.47 to 0.51 | .289 | 78.9% |
| Omitting Abedelmalek et al., 2022 | -0.20 | -0.50 to 0.10 | .151 | 36.8% |
| Omitting Chae et al., 2022 | -0.52 | -1.50 to 0.45 | .246 | 79.4% |
| Omitting Staiano et al., 2018 | -0.43 | -1.40 to 0.54 | .330 | 77.8% |
| Omitting Staiano et al., 2017 | -0.58 | -1.48 to 0.32 | .173 | 76.0% |
| Omitting Park et al., 2015 | -0.53 | -1.49 to 0.44 | .239 | 79.7% |
| Omitting Adamo et al., 2010 | -0.50 | -1.47 to 0.47 | .261 | 79.9% |
| Omitting Murphy et al., 2009 | -0.52 | -1.49 to 0.45 | .249 | 79.8% |
| Omitting Lu et al., 2025 | -0.54 | -1.51 to 0.43 | .229 | 78.9% |

Abbreviations: CI, confidence interval; I², proportion of variability attributable to heterogeneity.

**Triglycerides**

Hedges g ranged from -0.22 to -0.07; every 95% CI crossed the null. Each row reports the random-effects model after omitting the named study. Negative Hedges g values favor exergaming.

| **Omitted study** | **Hedges g** | **95% CI** | **P value** | **I²** |
| --- | --- | --- | --- | --- |
| Omitting Wang et al., 2025 | -0.07 | -0.43 to 0.28 | .643 | 41.3% |
| Omitting Abedelmalek et al., 2022 | -0.09 | -0.30 to 0.13 | .374 | 0% |
| Omitting Chae et al., 2022 | -0.22 | -0.60 to 0.16 | .211 | 46.0% |
| Omitting Staiano et al., 2018 | -0.17 | -0.57 to 0.23 | .340 | 55.0% |
| Omitting Staiano et al., 2017 | -0.22 | -0.57 to 0.14 | .190 | 47.3% |
| Omitting Park et al., 2015 | -0.17 | -0.56 to 0.22 | .337 | 55.0% |
| Omitting Adamo et al., 2010 | -0.16 | -0.55 to 0.22 | .348 | 55.0% |
| Omitting Murphy et al., 2009 | -0.18 | -0.57 to 0.21 | .311 | 54.7% |
| Omitting Lu et al., 2025 | -0.19 | -0.60 to 0.21 | .291 | 53.9% |

Abbreviations: CI, confidence interval; I², proportion of variability attributable to heterogeneity.
